# Being My Own Companion in Times of Social Isolation – A 14-Day Mobile Self-Compassion Intervention Improves Stress Levels and Eating Behavior

**DOI:** 10.3389/fpsyg.2020.595806

**Published:** 2020-10-09

**Authors:** Rebekka Schnepper, Julia Reichenberger, Jens Blechert

**Affiliations:** Division of Health Psychology and Centre for Cognitive Neuroscience, Department of Psychology, Paris-Lodron-University of Salzburg, Salzburg, Austria

**Keywords:** self-compassion, emotional eating, COVID-19, isolation, intervention study, stress reduction, ecological momentary intervention

## Abstract

The worldwide spread of the coronavirus disease (COVID-19) and the resulting lockdown has affected the whole world and the maintenance of healthy eating behavior might be an additional challenge. Self-compassion (SC) interventions emphasize not only treating oneself in a caring way regarding personal weaknesses, e.g., diet lapses, but also the recognition of shared human suffering. Thus, self-compassion might be particularly valuable during the current worldwide crisis due to COVID-19. In this study, *N* = 65 participants that wanted to lose weight or develop a healthier eating behavior were randomized to either a 14-day self-compassion intervention arm or a waitlist control arm. The intervention consisted of daily journaling exercises and meditations *via* smartphone with a focus on improving eating behavior. Before and after the intervention phase, questionnaires on self-compassion, eating, dieting, health behavior, stress, and emotion regulation were completed and body weight was determined. Participants in the treatment arm (*n* = 28) showed an increase in self-compassion, a decrease in perceived stress, eating in response to feeling anxious, and, on trend level, body mass index (BMI). Changes in self-compassion fully mediated changes in stress. No such effects were found in the waitlist control group (*n* = 29). Thus, self-compassion might help to maintain well-being and healthy eating habits in times of increased stress and isolation. Future studies should replicate these findings outside of the COVID-19 crisis and test the effect of self-compassion in samples with eating disorders or weight problems.

## Introduction

At the beginning of 2020, governments all over the world passed laws to curb the spread of the coronavirus disease (COVID-19), caused by the severe acute respiratory syndrome coronavirus 2 (SARS-CoV-2). This new virus is highly contagious and became a global pandemic within weeks. Groups at risk for a severe course are older people or people with preexisting chronic diseases like diabetes or autoimmune disorders. However, also in healthy adults, fatal cases with respiratory or cardiac failure as a frequent cause of death occurred (Beeching et al., 2020). Due to the restrictions that lasted at least 2 months in most of the countries, public life halted – educational institutions, restaurants/bars/cafés, cultural and sports facilities, and non-essential shops were closed, events were canceled, and gatherings with people from different households were forbidden. During the peak of daily infections and deaths, more than half of the world population was on some sort of lockdown (Sandford, 2020).

The pandemic confronted people with various fears: contracting COVID-19 themselves (Fitzpatrick et al., 2020), job loss due to economic recession, and having to postpone long-term plans, like a change in occupation, place of residence, or getting a degree in time. Further, social isolation during lockdown can result in loneliness, a worsening of pre-existing mental illnesses, and in increased anxiety and depression in populations that experience additional strain, e.g., health care workers and parents (Holmes et al., 2020). A review that investigated the psychological impact of being in quarantine identified a longer duration, fear of getting infected, frustration, boredom, inadequate basic and medical supplies, inadequate information on the current situation, financial loss, and stigma when being quarantined due to exposure to the virus as stressors (Brooks et al., 2020). In a Swiss general population study, half of the participants reported an increase in stress, and 57% of the participants had an increase in depression scores and in anxiety levels (de Quervain et al., 2020). In a Spanish sample, especially women, people with a history of mental illness, and people experiencing symptoms of COVID-19 themselves or in close relatives reported a worsening of mental health (González-Sanguino et al., 2020). Thus, it is important to provide practical advice, support, and coping strategies for managing boredom, loneliness, and stress (Brooks et al., 2020).

Isolation and quarantine might also affect health behavior like physical activity or eating negatively. To illustrate, restrictions during the lockdown period made it more difficult to remain physically active (Pinto et al., 2020), which again can have a negative impact on mental health (Lippi et al., 2020). Further, stress (Adam and Epel, 2007), negative emotions (Konttinen et al., 2010), and boredom (Havermans et al., 2015) can cause an increase in food cravings and a preference for high-calorie “comfort food.” Besides, loneliness and social isolation have been related to the occurrence of binge eating (Mason et al., 2016) and the risk of developing an eating disorder (Levine, 2012). Indeed, there is initial evidence that the COVID-19 lockdown had a negative impact on eating behavior. In an international survey, participants reported unhealthier eating habits, which might be partly due to a higher availability of unhealthy food as a consequence of stockpiling to avoid potential shortages of food (Ammar et al., 2020). In an Italian sample, half of the participants reported eating more during lockdown, especially comfort food, and 19.5% reported weight gain. Participants attributed this to an increase in anxiety, boredom, and stress (Scarmozzino and Visioli, 2020). Thus, measures might be needed that promote healthy eating directly and also support coping with its predecessors like stress and isolation to prevent negative consequences of restrictions due to COVID-19 (Lippi et al., 2020). In this context, self-compassion (SC) might play an important role, especially for vulnerable populations that were already concerned about their diet or weight prior to the lockdown.

Self-compassion is a concept that recently attracted a lot of interest. It can be defined by having a kind, non-judgmental attitude towards the own self, especially regarding perceived weaknesses (Neff, 2003b). Through SC, reflecting on these weaknesses is not avoided, but rather dealt with in a compassionate, gentle attitude and the aim to identify and fulfill own personal needs – similarly how one would treat a good friend in such a situation (Neff, 2003b). According to Neff (2003b), self-compassion entails three interrelated components: First, self-kindness, i.e., being gentle toward oneself in the face of failure and inadequacies (vs. self-judgment due to frustration with these shortcomings), second, common humanity, i.e., recognizing that everyone experiences suffering (vs. self-isolation, i.e., getting absorbed in an egocentric perspective on one’s own problems). Third, non-judgmental mindfulness/present moment awareness (vs. over-identification with negative feelings). Studies show that higher SC is associated with less worrying (Keng et al., 2012), as well less anxiety and depression symptoms (Van Dam et al., 2011). In addition, SC correlates with more adaptive coping in response to negative emotions and negative events (Leary et al., 2007). Thus, SC might aid in protecting against the abovementioned negative consequences of social isolation due to the COVID-19 lockdown.

With regard to eating behavior and body weight, SC has shown potential to improve factors that are not addressed by traditional diets, including body image and disordered eating (Rahimi-Ardabili et al., 2018). It also helps to reduce unhealthy eating styles, including restrictive eating (Adams and Leary, 2007) and binge eating (Pinto-Gouveia et al., 2019), which are risk factors for the later development of an eating disorder. Further, it might facilitate mindful eating by making individuals more receptive for mindfulness interventions (Mantzios and Wilson, 2015). Self-kindness instead of self-isolation has been identified as possible pathways how self-compassion can prevent binge eating (Webb and Forman, 2013). These abilities might be especially helpful in times of increased distress and isolation during the COVID-19 pandemic. Although a review showed beneficial effects of SC on eating behavior, body image, and weight loss (Rahimi-Ardabili et al., 2018), previous studies suffered from several limitations like failing to include a control group (Pinto-Gouveia et al., 2019), combining SC with other helpful components like mindfulness, yoga, and psychoeducation, or only assessing short-term effects. Furthermore, no study has yet explored SC interventions in the context of a global crisis as the current one, despite their high applicability to fundamental and existential threats.

In this study, participant who wanted to lose weight or change their eating behavior received a 2-week, smartphone-based self-compassion intervention. Effects were compared to a waitlist control group. We hypothesized that first; self-compassion would increase in the intervention group (IG) compared to baseline, while no change would be evident in the waitlist group (WG). Second, we expected a positive effect of the intervention on stress experience related to COVID-19 restrictions. Third, we hypothesized that the intervention would help participants to improve their eating behavior and reduce their body mass index (BMI). For the second and third hypothesis, we explored whether changes in self-compassion mediate possible changes in stress, eating behavior, and BMI and if this relation was different between the two groups.

## Materials and Methods

### Participants

We aimed for a sample size of 80 participants (40 participants per condition), based on a power (1-ß) of 0.80, *α* = 0.05, and a medium effect size of *f* = 0.25 in a repeated measures ANOVA with within-between interaction, two groups, and two time points. This estimation took a possible dropout rate of 15% into account. However, since the duration of the lockdown phase was unclear and the intervention lasted 14 days, we further took into consideration how many participants could be tested in a short period of time when calculating the sample size. Data collection was stopped when signs pointed toward the lockdown measures being eased. The final sample consisted of *N* = 57 participants who were recruited *via* newspaper articles, social media, and university newsletters between March and May 2020 when lockdown restrictions were stepwise increased in Austria and Germany.^1^ After expressing interest, they first filled out an online questionnaire to determine whether they met inclusion and did not meet exclusion criteria. Inclusion criteria were being fluent in German, experiencing impairment in daily life due to the COVID-19 lockdown (i.e., not going to work as usual), and the goal to lose weight or to develop a healthier eating behavior. Exclusion criteria were a lifetime eating disorder and a current pregnancy or breastfeeding. All participants received an individual feedback based on their data; psychology students additionally had the possibility to receive five study credits (26.3% of the final sample). In the final sample, 64.9% of the participants were students, 22.8% were employees, and 12.3% had other occupations. The most frequent main reason for participating was the wish to lose weight (40%), followed by wanting to eat healthier (39%), wanting to have more regular meals (12%), and wanting to improve their emotional eating patterns (9%). See Table 1 for further sample characteristics.

**Table 1:** Baseline characteristics of the final sample divided by intervention group (IG) and waitlist control group (WG). Values show means (*M*) and standard deviations (*SD*) or number of individuals (*N*) and percentage (%).

	IG (*N* = 28)	WG (*N* = 29)	
Variable	*M* (*SD*)	*M* (*SD*)	*p*
Age (in years)	27.0 (7.52)	31.0 (14.0)	0.191
Body mass index (in kg/m^2^)^*^	22.1 (2.97)	23.9 (4.44)	0.087
Years of education	17.0 (2.63)	16.5 (2.85)	0.479
Self-compassion	2.92 (0.56)	2.94 (0.67)	0.834
Perceived stress	21.0 (6.35)	19.0 (6.35)	0.232
Sadness eating	3.24 (0.61)	3.39 (0.65)	0.372
Anxiety eating	2.81 (0.66)	2.62 (0.63)	0.261
Anger eating	2.87 (0.53)	2.87 (0.60)	0.987
Overall emotional eating	2.97 (0.44)	2.96 (0.52)	0.906
**Variable**	***N* (%)**	***N* (%)**	
Sex (female)	25 (89.3)	23 (79.3)	0.306
Currently dieting	18 (64.3)	14 (48.3)	0.227

* One participant was excluded from analyses due to being an outlier in BMI (BMI > 40; > 2*SD*), which lead to BMI baseline differences.

### Procedure

**Figure 1:**
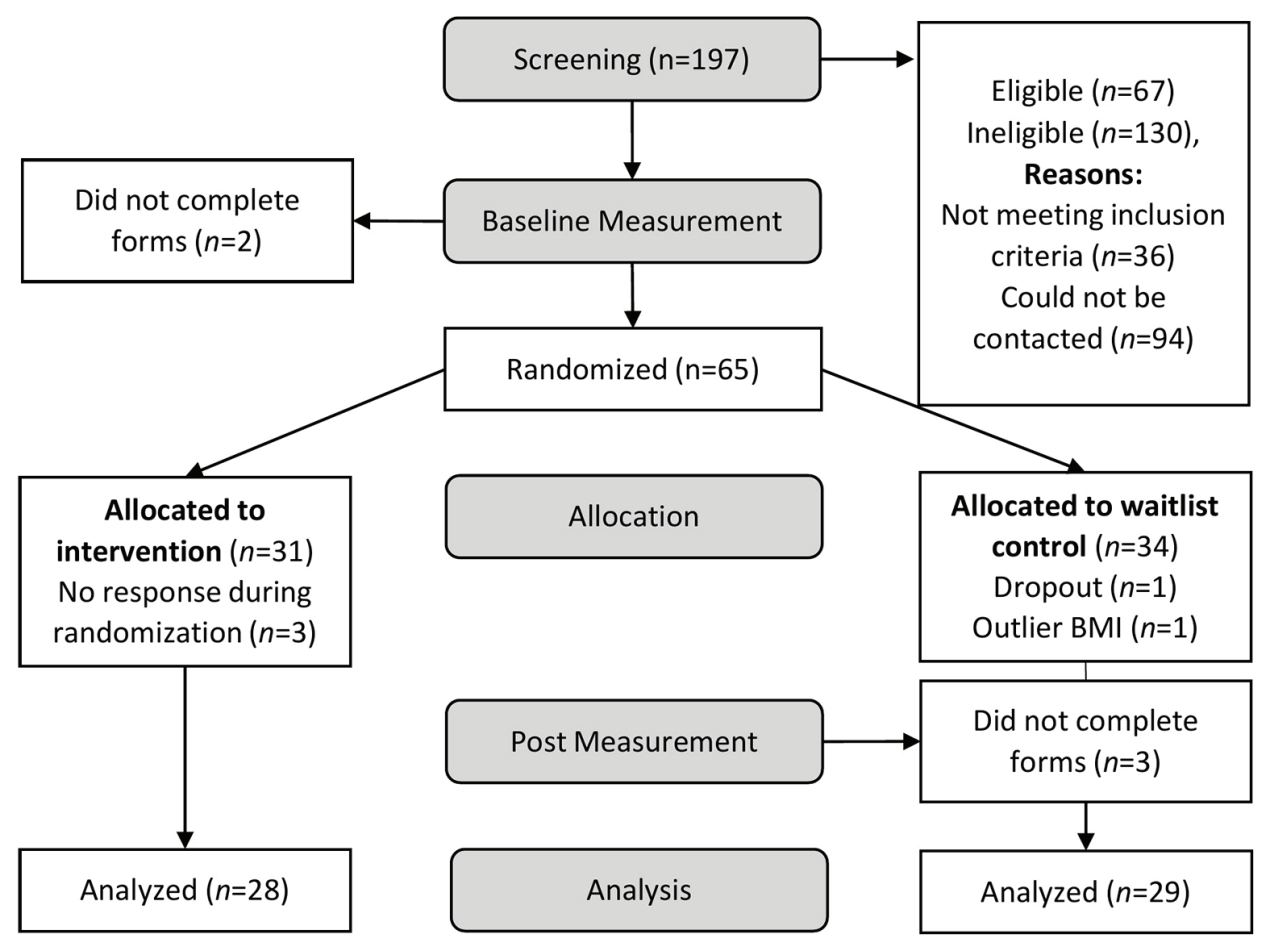
Phases of the study design and number of participants who completed each phase. Participants that met eligibility criteria were invited to fill out the baseline questionnaire.

Participants that met inclusion criteria received an informed consent form with the instruction to sign it and send it back *via* e-mail. Further, they received a link to the baseline questionnaire, which included demographic information as well as questionnaires on self-compassion, eating, dieting, health behavior, stress, emotion regulation, and a virtual food rating task. After completing the baseline questionnaire, participants were randomly allocated to the IG or WG, using a randomization scheme created with the website randomizer.org. Then, they received login information for the app PsyDiary and installation instructions *via* a telephone call. The app was used during the 14-day intervention to provide the self-compassion exercises for the IG. Further, both groups answered end-of-the-day questions on self-compassion, mindfulness, mood, eating behavior, and experienced consequences of the lockdown (results reported elsewhere). Daily notifications reminded participants to do the self-compassion exercise and to answer the questionnaire. Afterward, participants completed a post questionnaire similar to baseline. Upon completion, the WG received the intervention. The ethics committee of the University of Salzburg, Austria approved of the study. See Figure 1 for a flowchart of the study.^2^

### Self-Compassion Intervention

The 2-week SC intervention that aimed to increase SC was mostly inspired by material provided by Dr. Kristin Neff (2020). It consisted of three different meditations and eight different journaling exercises in alternating order. Both journaling exercises and meditations have been found to increase self-compassion and thereby assist in weight loss (Mantzios and Wilson, 2014). Exercises were adapted to the theme of improving one’s eating behavior. In this context, participants learned to be mindful and less critical about negative emotions, but rather see them as a part of being human. When starting the intervention, participants were instructed to follow the structure of the 2-week plan that indicated which exercise should be done on each day. They were also encouraged to start a SC journal and to continue doing the exercises over the course of multiple weeks. The journaling exercises covered different, related topics: writing a letter about a perceived weakness regarding eating behavior to oneself from the perspective of a loving friend, reflecting on how participants would treat a friend in a similar situation, exploring the participants’ self-criticism when trying to improve their eating behavior, and finding alternative and less critical ways to motivate themselves. Further, they learned how to treat themselves kindly when experiencing food cravings, to recognize that unhealthy eating is not self-compassionate, and to find alternatives to reward themselves. The meditations contained compassionate breathing exercises, SC affirmations and soothing touch. Each meditation repeated in the 2nd week, while each journaling exercise was only done once.

### Measures

#### Self-Compassion Scale, German Version

The German 26-item version (Hupfeld and Ruffieux, 2011) of the original scale (Neff, 2003a) consists of six subscales that assess three positive components of SC as well as three negative counterparts: Self-kindness (as opposed to self-judgment), common humanity (as opposed to isolation), and mindfulness (as opposed to over-identification). In previous studies, subscales were highly intercorrelated and best explained by an underlying construct of general self-compassion (Neff, 2003a; Hupfeld and Ruffieux, 2011). Participants indicate how they typically act toward themselves in difficult times from 1 = “almost never” to 5 = “almost always.” An example item for self-kindness is “I’m kind to myself when I’m experiencing suffering,” an example for common humanity is “When I feel inadequate in some way, I try to remind myself that feelings of inadequacy are shared by most people,” and an example for mindfulness is “When I’m feeling down I try to approach my feelings with curiosity and openness.” After reverse coding negative items, a sum score for SC can be calculated. Internal consistencies in the present study were Cronbach’s α = 0.910 for self-kindness, *α* = 0.769 for common humanity, *α* = 0.706 for mindfulness, *α* = 0.726 for self-judgment, *α* = 0.765 for isolation, *α* = 0.606 for over-identification, and *α* = 0.919 for the overall scale at baseline and Cronbach’s α = 0.859 for self-kindness, *α* = 0.864 for common humanity, *α* = 0.729 for mindfulness, *α* = 0.855 for self-judgment, *α* = 0.744 for isolation, *α* = 0.640 for over-identification, and *α* = 0.927 for the overall scale after the intervention phase (Hupfeld and Ruffieux, 2011).

#### Perceived Stress Scale, German Version

The German version (Klein et al., 2016) of the perceived stress scale (PSS; Cohen et al., 1983) investigates the experience of psychological stress in the past month. It consists of 10 items (e.g., “In the last month, how often have you been upset because of something that happened unexpectedly?”) which are answered on a scale from 1 = “never” to 5 = “very often.” In this sample, stress levels were considerably higher than in previous general population samples (Cohen et al., 1983; Klein et al., 2016). Internal consistencies in the present study were Cronbach’s α = 0.834 at baseline and Cronbach’s α = 0.861 after the intervention phase (Klein et al., 2016).

#### Salzburg Emotional Eating Scale

The Salzburg emotional eating scale (SEES; Meule et al., 2018) assesses changes in eating behavior in response to four emotional states (happiness eating, sadness eating, anger eating, and anxiety eating). It consists of 20 items (e.g., “When I am worried, …”), which are rated from 1 = “I eat much less than usual” to 5 = “I eat much more than usual.” In this study, we included an overall scale of eating in response to negative emotions (i.e., sadness, anger, and anxiety), and each negative subscale separately. Internal consistencies in the present study were Cronbach’s α = 0.826 for sadness eating, *α* = 0.845 for anger eating, *α* = 0.853 for anxiety eating, and *α* = 0.878 for overall emotional eating at baseline and Cronbach’s α = 0.854 for sadness eating, *α* = 0.857 for anger eating, *α* = 0.813 for anxiety eating, and *α* = 0.887 for overall emotional eating after the intervention phase (Meule et al., 2018).

#### Body Mass Index

At baseline and post measurement, participants were asked to upload a photo of a scale showing the participants body weight.^3^ This information was then used to analyze changes in BMI.

### Statistical Analysis

Using SPSS 26 (IBM, 2019), independent *t*-tests were conducted to test for baseline differences between WG and IG. Further, 2 × 2 (Group × Time) mixed ANOVAs were computed to test the pre-specified hypotheses on changes in outcome variables between pre and post measurement, and whether the intervention would interact with this change. Eta squared was calculated as an estimate for effect sizes, with *η*^2^ > 0.01 indicative of a small effect, *η*^2^ > 0.06 of a medium effect, and *η*^2^ > 0.11 of a large effect. For further analyzing the intervention effect on the other outcome variables, an SC change score was computed and grand mean centered. The PROCESS 3.5 macro for SPSS (Hayes, 2013) was used to build a mediation model with groups as a predictor for change scores of stress, BMI, and eating behavior, and the SC change score as a mediator.

## Results

### Intervention Adherence

On average, participants in the IG reported that they completed an SC exercise on 11.5 of the 14 days (*SD* = 2.27). The exercises were estimated to be moderately helpful (*M* = 55.1 on a continuous scale from 0 to 100), with no difference between meditations and journaling exercises, *p* = 0.125. With specific regard to the current lockdown situation, the exercises were somewhat helpful (*M* = 43.3 on a continuous scale from 0 to 100). Here, participants reported meditations to be more useful, *p* = 0.019.

### Intervention Effects

#### Self-Compassion Scale

**Figure 2:**
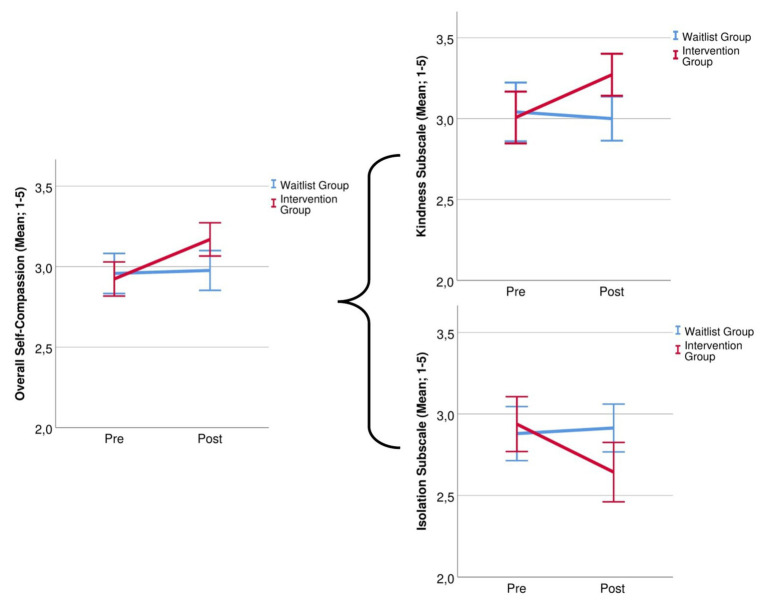
Group changes in overall self-compassion and for the subscales self-kindness and isolation before and after the 14-day intervention.

The overall SC score showed a main effect of Time, *F*(1,55) = 7.26, *p* = 0.009, *η*^2^ = 0.117, that was moderated by a Group × Time interaction, *F*(1,55) = 5.36, *p* = 0.024, *η*^2^ = 0.089. *Post hoc* tests showed that SC in the IG was higher after completing the intervention, *t*(27) = −3.10, *p* = 0.004, while it did not change in the WG, *t*(28) = −0.32, *p* = 0.754. Further, there was a significant Group × Time interaction for the subscales Self-kindness, *F*(1,55) = 4.59, *p* = 0.037, *η*^2^ = 0.077 and Isolation, *F*(1,55) = 4.33, *p* = 0.042, *η*^2^ = 0.073. Self-kindness in difficult situations increased, *t*(27) = −2.66, *p* = 0.013, and feelings of isolation in response to failure and negative mood decreased, *t*(27) = 2.74, *p* = 0.011, with no such effect in the WG, both *p*s > 0.689. There was a main effect of Time for Over-Identification, *F*(1,55) = 4.15, *p* = 0.046, *η*^2^ = 0.070, and Humanity, *F*(1,55) = 4.47, *p* = 0.039, *η*^2^ = 0.075, but no interaction with group. No significant main effects or interactions were found for Mindfulness, Self-judgment, all *p*s > 0.105. See Figure 2 for group differences in SC.

#### Perceived Stress Scale

**Figure 3:**
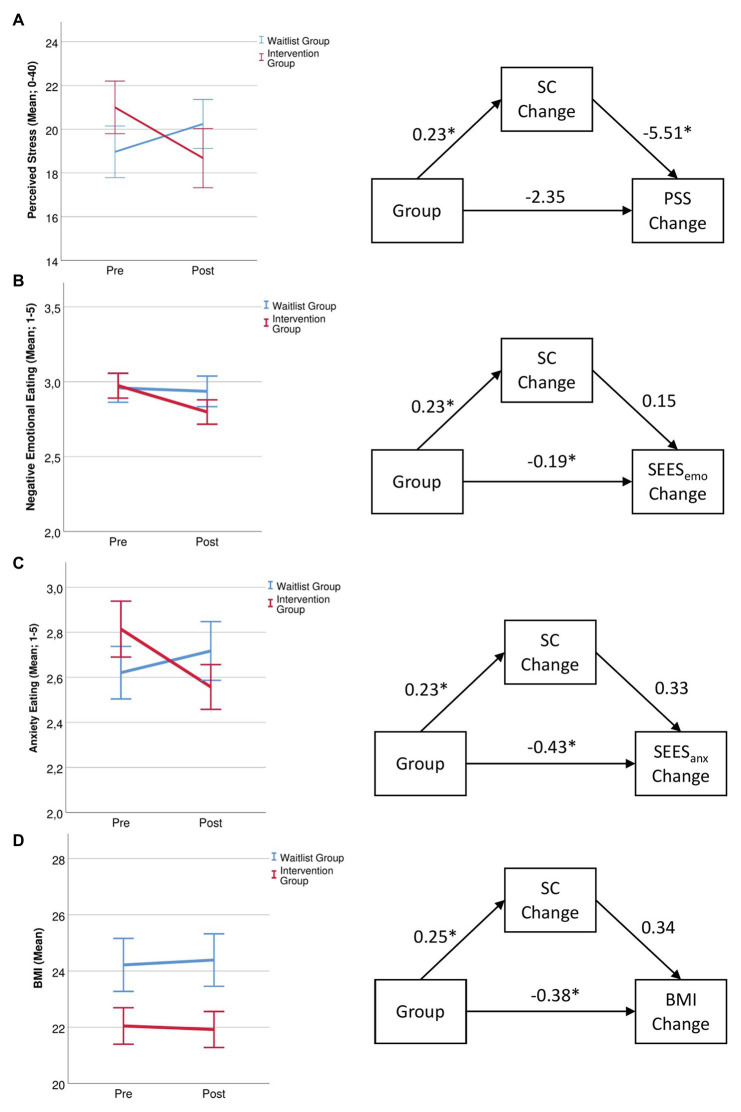
Changes in **(A)**, perceived stress, **(B)**, eating in response to negative emotions, **(C)**, eating in response to anxiety, and **(D)**, body mass index (BMI) for the waitlist and intervention group before and after the 14-day intervention and mediating role of self-compassion for these changes with correlation coefficients. ^*^ = *p* < 0.05, SC, self-compassion; PSS, perceived stress scale; SEES_emo,_ Salzburg emotional eating scale (overall emotional eating); SEES_anx,_ Salzburg emotional eating scale (anxiety eating subscale); and BMI, body mass index.

A Group × Time interaction, *F*(1,55) = 5.70, *p* = 0.020, *η*^2^ = 0.094 pointed to different time courses in the two groups. Figure 3A indicates that perceived stress decreased in the IG while it increased in the WG.

#### Salzburg Emotional Eating Scale

Analyzing the overall negative emotions scale revealed a main effect of time, *F*(1,55) = 6.89, *p* = 0.011, *η*^2^ = 0.111, that was moderated by a Group × Time interaction, *F*(1,55) = 4.08, *p* = 0.048, *η*^2^ = 0.069. Overall emotional eating decreased in the IG, *t*(27) = 2.89, *p* = 0.008, and did not change in the WG, *t*(28) = 0.50, *p* = 0.618 (Figure 3B). For the subscale anxiety eating, there was a Group × Time interaction, *F*(1,55) = 7.73, *p* = 0.007, *η*^2^ = 0.123. The IG ate less in response to anxiety, *t*(27) = 2.44, *p* = 0.021, while no change occurred in the WG, *t*(28) = −1.33, *p* = 0.195 (Figure 3C). For anger eating, there was only a main effect of Time, *F*(1,55) = 4.01, *p* = 0.050, *η*^2^ = 0.068, but no interaction, *p* = 0.586. Sadness eating did not show any main effects or interactions, all *p*s > 0.135.

#### Body Mass Index

Analyzing changes in BMI for the 81% of participants who were able to provide a photo of their body weight showed a trend Group × Time interaction, *F*(1,44) = 3.81, *p* = 0.057, *η*^2^ = 0.080. *Post hoc* tests showed a significant group difference at post measurement, *F*(1,47) = 4.58, *p* = 0.038, *η*^2^ = 0.089. Figure 3D shows that while BMI decreased in the IG, it increased in the WG.

### Mediation Analysis

#### Perceived Stress Scale

Analyzing the PSS change score in a mediation model showed a direct effect of group on changes in SC, *b* = 0.23, *t*(55) = 2.32, *p* = 0.024, and a direct effect of changes in SC on changes in perceived stress, *b* = −5.51, *t*(54) = −2.82, *p* = 0.007. The direct path of group on changes in perceived stress was not significant, *b* = −2.35, *t*(55) = −1.58, *p* = 0.120 (Figure 3A).

#### Salzburg Emotional Eating Scale

Analyzing change scores in a mediation model showed a direct effect of group on changes in SC, *b* = 0.23, *t*(55) = 2.32, *p* = 0.024 There was a direct effect of group on overall emotional eating, *b* = −0.19, *t*(54) = 1.41, *p* = 0.022, but no direct effect of SC changes on overall emotional eating, *b* = 0.15, *t*(54) = 1.41, *p* = 0.165 (Figure 3B). Group also had a direct effect of changes in anxiety eating, *b* = −0.43, *t*(54) = −3.29, *p* = 0.002, while changes in SC only had a direct effect on anxiety eating on a trend level, *b* = 0.33, *t*(54) = 1.92, *p* = 0.060 (Figure 3C).

#### Body Mass Index

Analyzing changes in BMI for the 81% of participants who were able to provide a photo of their body weight showed a trend Group × Time interaction, *F*(1,44) = 3.81, *p* = 0.057, *η*^2^ = 0.080. *Post hoc* tests showed a significant group difference at post measurement, *F*(1,47) = 4.58, *p* = 0.038, *η*^2^ = 0.089. Figure 3D shows that while BMI decreased in the IG, it increased in the WG.

## Discussion

In this study, we applied a mobile 2-week SC intervention to individuals that wanted to reduce their weight or improve their eating behavior during the COVID-19 lockdown. Intervention effects were compared to a waitlist control group, which only answered daily questionnaires without completing an exercise. As expected, the IG showed increases in SC, which was especially visible in an increase in self-kindness and a decrease in self-isolation. In the context of the lockdown, experiencing less isolation might be particularly beneficial for mental well-being (Liu et al., 2020). Further, perceived stress during lockdown decreased in the IG. This is in line with recent studies that showed the positive effect of a mindfulness intervention on anxiety and sleep quality during the COVID-19 lockdown (Zheng et al., 2020). Mediation analyses showed that a reduction in stress was due to increases in SC in the IG, showing that training SC can be a helpful tool to acquire stress coping skills. This is remarkable because reported stress in this sample was almost as high as in a sample of patients treated for work-related stress and mood disorders (Glasscock et al., 2018), which shows the detrimental effect that the lockdown policies had on mental health.

The intervention also had a positive effect on emotional eating: the IG reported less eating in response to negative emotions, especially in response to anxiety. As the lockdown and uncertainties related to the spread of the virus has the potential to increase anxieties, which has been shown to negatively impact eating behavior (Scarmozzino and Visioli, 2020), SC can pose a protective factor against the establishment of unhealthy eating habits. Further, BMI trend effects are in line with the previously reported protective role of SC against binge eating disorder (Pinto-Gouveia et al., 2019). However, a lack of significant results might also indicate a conflicting effect of self-compassion: when feeling psychologically unwell, physiological long-term health might not be prioritized. Consequently, self-kindness might lead to self-indulgence and an unhealthy but comforting snack might be permitted (Mantzios and Egan, 2017; Egan and Mantzios, 2018). If future studies with a higher power show similar, more robust effects, this would be especially valuable as studies show the potential weight gain during the lockdown (e.g., Di Renzo et al., 2020). Unfortunately, not all participants were able to track weight changes since they did not own a scale, and due to the lockdown could not go elsewhere to weigh themselves. While other studies found a direct positive effect of SC components on eating behavior (Webb and Forman, 2013), mediation analysis showed no direct effect of SC on emotional eating or weight. Considering the increase in stress levels during the lockdown period (de Quervain et al., 2020), which were reported to be a cause for unhealthy eating behavior (Scarmozzino and Visioli, 2020), the decrease in perceived stress might have helped to eat more balanced and less in response to negative emotions. Again, a larger sample might help to clarify these effects that were significant on a trend level.

Besides the rather small group size, it has to be noted that although compliance to do an SC exercise each day was very high, the perceived helpfulness of the SC exercises can be improved. More guidance during the intervention phase, personalization of the training plan, and individualized diet or weight goals during the intervention period might help to increase the effect of the intervention. A long-term follow-up might help to determine the temporal stability of effects. In the waitlist group, participants filled out daily questionnaires on SC, eating behavior, mindfulness, and mood, which were needed for comparing EMA data in the two groups. However, this could have drawn the participants’ attention to these topics, thus creating an attenuated intervention effect. Lastly, we did not preregister our hypotheses due to the limited amount of time in the lockdown situation. Future studies are planned to overcome these shortcomings and replicate findings.

Despite these limitations, this study has various strengths. Following recommendations of Rahimi-Ardabili et al. (2018), we tested the effect of an SC intervention and compared it to a waitlist control group, thus acquiring longitudinal and causal effects instead of mere correlational data. Effect sizes were either medium or large, highlighting the potential of our intervention. Further, we were able to apply the SC intervention in a highly stressful and potentially threatening time that affected everyone to some degree. Previous studies showed the risk for weight gain especially in vulnerable populations like individuals with obesity (Almandoz et al., 2020). Although the present study did not explicitly target overweight or obese individuals, we focused on individuals with an interest in weight loss who might face similar challenges during the lockdown. As face-to-face meetings were hardly possible during the COVID-19 lockdown, the benefit that participants drew from the intervention is of high value. Research calls for e-mental health technologies to provide necessary interventions during the COVID-19 lockdown (Wind et al., 2020). The use of a mobile smartphone app and the intervention and installation instruction *via* phone further made the intervention feasible as well as cost and time saving during the lockdown situation. In the future, it might also be used as an add-on to existing therapies. In conclusion, the present study provides promising data on the positive effect of SC interventions, which should be transferred to regular daily life after the lockdown and to other vulnerable groups, e.g., individuals with obesity or an eating disorder.

## Data Availability Statement

The raw data supporting the conclusions of this article will be made available by the authors, without undue reservation.

## Ethics Statement

The studies involving human participants were reviewed and approved by Ethics Committee of the University of Salzburg, Austria. The patients/participants provided their written informed consent to participate in this study.

## Author Contributions

RS designed and conducted the study, did the statistical analyses, and drafted the manuscript. JR helped with the design of the study and contributed to the final draft. JB helped with the design of the study, acquired funding, and contributed to the final draft. All authors contributed to the article and approved the submitted version.

### Conflict of Interest

The authors declare that the research was conducted in the absence of any commercial or financial relationships that could be construed as a potential conflict of interest.

## Footnotes

^1^Due to the unpredictability of the lockdown duration, we did not define a precise sample size, but aimed to include at least *n* = 40 participants per condition. Our final sample is slightly below this aim, but comparable to other studies that applied a self-compassion training (Adams and Leary, 2007; Pinto-Gouveia et al., 2019).

^2^Analyzing all participants that responded to the randomization (*n* = 62) in an intention-to-treat approach did not change pattern or significance of the Time × Group interactions: overall SC: *p* = 0.013; SC Self-kindness subscale: *p* = 0.026; SC isolation subscale: *p* = 0.027; PSS: *p* = 0.017; SEES (anxiety subscale): *p* = 0.005; BMI: *p* = 0.097.

^3^*n* = 5 in the IG and *n* = 6 in the WG did not own a scale and were not able to determine their weight due to the lockdown.
